# Oil Production and Groundwater Quality in the Edwards-Trinity Plateau Aquifer, Texas

**DOI:** 10.1100/tsw.2003.111

**Published:** 2003-11-25

**Authors:** Paul F. Hudak

**Affiliations:** Department of Geography and Environmental Science Program, University of North Texas, P.O. Box 305279, Denton, TX 76203-5279, USA

**Keywords:** Edwards-Trinity Plateau Aquifer, oilfield brine, groundwater monitoring

## Abstract

Chloride concentrations and chloride/bromide ratios from 198 water wells in the Edwards-Trinity Plateau Aquifer were compiled, mapped, and evaluated within the context of regional geology and land use. The study area occupies eight counties in west-central Texas, within which oil production and agriculture are predominant land uses. Samples from 49 wells had chloride concentrations above the 250 mg/l secondary drinking water standard, 22 samples had greater than 500 mg/l chloride, and 9 samples exceeded 1000 mg/l chloride. Of the 22 samples above 500 mg/l chloride, 10 had relatively low chloride/bromide ratios of less than 300, consistent with oilfield brine, and 2 had ratios above 2000, consistent with groundwater impacted by evaporite dissolution. The remaining ten samples had chloride/bromide ratios ranging from 300 to 2000, consistent with partial mixing of unimpaired groundwater with evaporite-laden water. There were no significant correlations between chloride concentration and well depth, inconsistent with contaminants originating at the land surface. Results of this study suggest that evaporite dissolution and oilfield brine locally impact the Edwards-Trinity Plateau Aquifer, but the problem is not regionally pervasive.

## INTRODUCTION

Large volumes of wastewater in the form of brine usually accompany oil and gas production. Oilfield brine is one of the principal pollutants of Texas aquifers[1]. High chloride concentrations and low chloride/bromide ratios (<300) commonly characterize groundwater impacted by oilfield brine. On average, oilfield brine contains 50,000 mg/l chloride, which along with other constituents of brine, can be toxic to crops, corrosive to metal, and unsafe to drink[1].

Historically, oilfield brine has been discharged to pits and gullies and sprayed onto roads. Currently, deep injection wells handle most of the oilfield brine generated in Texas. Both past and present oilfield brine disposal practices can contaminate groundwater.

.

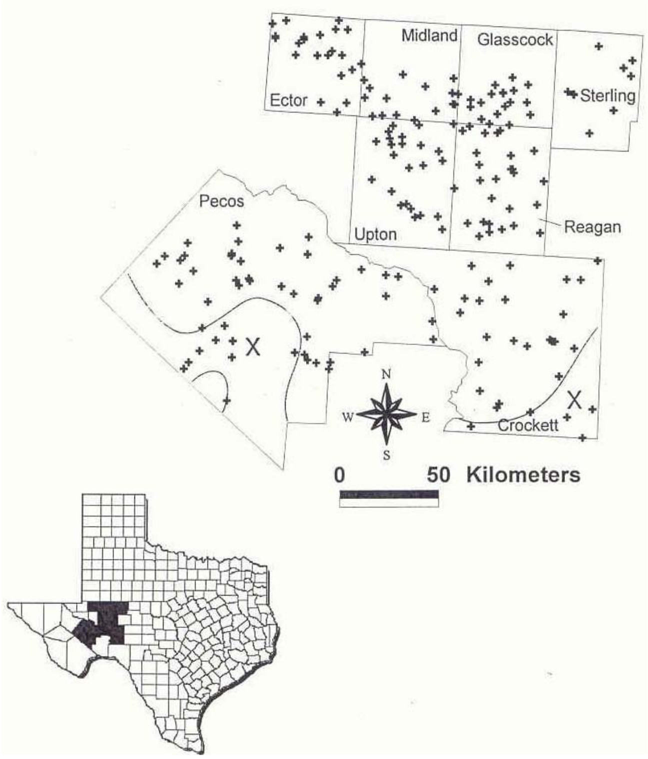

**FIGURE 1.** Study area and sampled water wells. X designates area sparsely occupied by oil wells[2].

Oilfield brine is a potential source of groundwater contamination in the Edwards-Trinity Plateau (ETP) aquifer of west-central Texas. The aquifer overlies vast and heavily tapped oil and gas reservoirs. The objective of this study was to map and evaluate distributions of chloride and chloride/bromide ratios from water wells in relation to oil production within an eight-county area of west-central Texas (Fig. 1)

## STUDY AREA

The study area covers eight counties in the rural, rocky plains of west-central Texas (Fig. 1). Oil production and agriculture are predominant land uses in the study area. Rangeland and cropland — primarily cotton, sorghum, and alfalfa — cover most of the region. Since the early 1900s, thousands of oil wells have been drilled in the study area (Table 1). Oil wells occupy each county of the study area, but only sparsely occupy portions of Pecos and Crockett Counties (Fig. 1).

In 1969, the Texas Railroad Commission prohibited using unauthorized pits to dispose of oilfield brine[4]. However, pits are still being used for drilling fluid and emergency saltwater storage. Potential routes through which oilfield brine can enter groundwater in the study area include leaking casings in production wells, leaking injection wells, upward migration through abandoned wells not plugged or capped, and surface infiltration from past dumping[4].

**TABLE 1**
**Oil and Injection Well Counts[3]**

| County | Producing Oil | Inactive | Injection |
|---|---|---|---|
| Ector | 5468 | 3093 | 1859 |
| Midland | 4127 | 663 | 159 |
| Glasscock | 1165 | 331 | 146 |
| Sterling | 1354 | 158 | 103 |
| Upton | 2038 | 1267 | 305 |
| Reagan | 3519 | 436 | 116 |
| Pecos | 2813 | 2311 | 554 |
| Crockett | 2136 | 788 | 188 |

The ETP Aquifer is the main source of groundwater in the study area. It supplies water for irrigation, municipal, industrial, livestock, and domestic uses[5]. The unconfined aquifer consists principally of shallow marine limestone, dolomite, and evaporites with secondary porosity (upper part) and interbedded fluvial sand, sandstone, and clay (lower part) deposited during the Cretaceous Period[1,6,7,8]. A potential source of dissolved solids in the ETP aquifer, evaporites also occupy underlying Triassic and Permian rocks and overlying alluvium along the Pecos River (northern boundary of Pecos County in Fig. 1)[7,9].

Precipitation is the main source of recharge to the ETP Aquifer. Annual precipitation and recharge in the study area average about 19 and 0.5 in., respectively[6,8]. Groundwater flows generally southeastward, locally converging toward the Pecos River, and discharges to wells, streams, and springs. Average depth to groundwater and saturated thickness of the ETP Aquifer in the study area are approximately 60 and 80 m, respectively[6,7]. Water wells in the study area yield an average of approximately 8 l/s[5].

## METHODS

Water quality and well data were obtained from 198 wells monitored by the Texas Water Development Board (Fig. 1). The wells were used for domestic supply (41 wells), irrigation (34 wells), stock (79 wells), public supply (22 wells), industry (11 wells), fire (2 wells), dewatering (1 well), and monitoring (8 wells). Well depth (163 observations) ranged from 18.0 to 396.3 m, with a median of 76.2 m. Wells were pumped until temperature, conductivity, and pH stabilized. Samples were taken directly from each well, filtered, preserved, and delivered to an analytical laboratory within 24 h. Analyses were completed using automated colorimetry or ion chromatography. Water quality data were collected from 1998 to 2003. The most recent sample was evaluated in wells with multiple sampling dates.

Chloride concentrations, chloride/bromide ratios, and well depth were compiled in Excel (Microsoft Corporation, Redmond, WA) and mapped with ArcView (Environmental Systems Research Institute, Redlands, CA). Summary and correlation statistics were computed with MINITAB (Minitab Incorporated, State College, PA).

## RESULTS AND DISCUSSION

Chloride concentrations ranged from 6.3 to 3735 mg/l, with a median of 96.2 mg/l (Table 2). Samples from 49 water wells exceeded the secondary drinking water standard of 250 mg/l[10]. Several observations were more than double the secondary standard: 22 exceeded 500 mg/l chloride and 9 exceeded 1000 mg/l chloride. Fig. 2 illustrates clusters of high chloride concentrations in Ector, Sterling, Reagan, and Pecos Counties.

**TABLE 2**
**Summary of Solute Concentrations**

| Solute | Number of Observations | Minimum | Maximum | Median |
|---|---|---|---|---|
| Chloride (mg/l) | 198 | 6.3 | 3735 | 96.2 |
| Chloride/bromide[a] | 180 | 21 | 3531 | 205 |

[a] Excluding 18 undefined ratios (bromide not detected): >91, >148, >148, >150, >163, >175, >199, >220, >220, >238, >248, >378, >396, >422, >858, >875, >890, >20000.

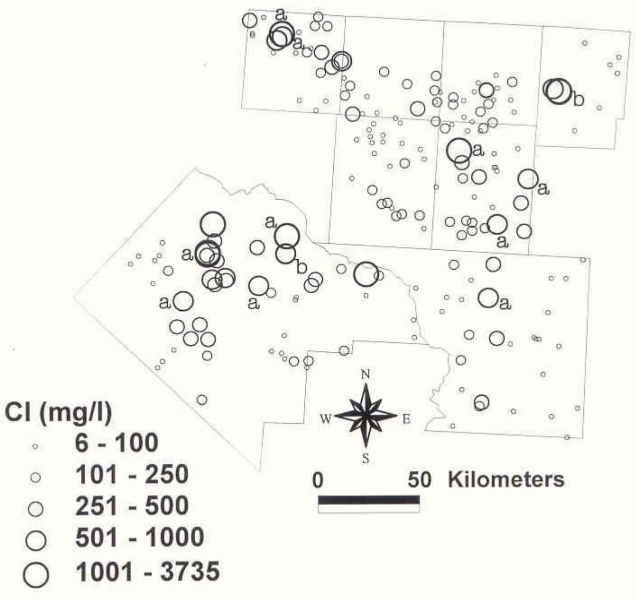

**FIGURE 2**. Map of chloride concentrations; a and b designate wells with >500 mg/l chloride and <300 or >2000 chloride/bromide ratios, respectively.

Chloride concentrations above 500 mg/l are generally uncommon in shallow groundwater. In the present study, such high concentrations were observed in six of eight counties, and chloride concentrations above 1000 mg/l were evident in Ector, Sterling, Reagan, and Pecos Counties. Considering predominant land use and geology within the study area, potential sources of elevated chloride in groundwater include oilfield brine, evaporite dissolution, and irrigation return flow.

In additon to elevated chloride, wells impacted by oilfiled brine would likely have low chloride/bromide ratios. Chloride/bromide ratios below 300 commonly characterize oilfield brine[11]. Of 22 wells with greater than 500 mg/l chloride, 10 had chloride/bromide ratios less than 300. Many of these water wells overlie oilfields (Figs.1 and 3).

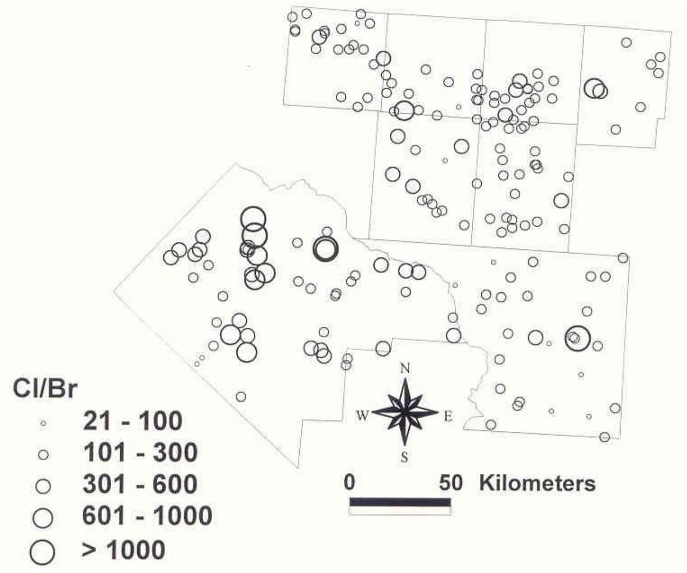

**FIGURE 3.** Map of chloride/bromide ratios (excluding undefined ratios in Table 2).

Potentially, dissolution of evaporite deposits could account for some of the high chloride/bromide observations. Natural evaporite dissolution brines usually have chloride/bromide ratios above 2000[11]. Two water samples, in Pecos and Sterling Counties, had greater than 500 mg/l chloride and chloride/bromide ratios exceeding 2000 (Figs. 2 and 3).

The remaining ten samples exceeding 500 mg/l chloride had chloride/bromide ratios from 300 to 2000. Mixing of unimpaired groundwater with evaporite-laden water, or mixing of brine with evaporite-laden water could account for these observations. That these observations occupy various parts of the study area is consistent with widespread evaporite deposits within and beneath the ETP Aquifer, and locally in overlying alluvium.

Evaporating irrigation water concentrates salts in soil. Subsequent rainfall or heavy irrigation may dissolve and carry constituents of these salts through the vadose zone. Thus irrigation return flow potentially contributes to chloride patterns outlined above. Observations of nitrate in groundwater in previous work[12] suggests local impacts from fertilized cropland in the study area. However, less than 5% of the study area is irrigated[1]. Moreover, if irrigation return flow exerted a major control on chloride in groundwater, shallow wells would likely have higher chloride concentrations. However, there was no significant correlation between chloride concentration and well depth in this study (rank correlation coefficient = 0.046). Similarly, Bush et al.[7] found only a weak relationship between pumping for irrigation and total dissolved solids concentrations in a broader region encompassing part of the study area.

Results presented above are consistent with groundwater impacted by evaporite dissolution and oilfield brine. Information presented in this study may be relevant to locating new water wells. Such wells should be located away from elevated chloride concentration clusters, but not necessarily deeply in the aquifer, as no trend for decreasing chloride concentration with depth was observed. The mapped data may also direct more intensive, localized investigations of groundwater quality to assess pollution sources and potential for remedial action, such as capping abandoned oil wells.

## CONCLUSION

Recent chloride and bromide data suggest evaporite dissolution and possibly oilfield brine account for clusters of high chloride levels in groundwater of the Edwards-Trinity Plateau Aquifer in west-central Texas. Nearly 50 of 198 water quality observations exceeded the secondary drinking water standard of 250 mg/l chloride. Over 20 water samples had chloride concentrations more than double the drinking water standard. Many of these observations also had chloride/bromide ratios consistent with oilfield brine or evaporite dissolution. Such high solute concentrations can render groundwater unfit for drinking and other uses. Results of this study may be useful for prioritizing local groundwater quality investigations to guide corrective action and placement of new water supply wells.

## BIOSKETCH

Dr. Paul F. Hudak is a Professor in the Department of Geography and Environmental Science Program at the University of North Texas. He received a BS in Geology from Allegheny College, MS in Geology from Wright State University, and PhD in Geography from the University of California, Santa Barbara. His research interests include groundwater monitoring and remediation, geologic hazards, and geoscience education.
